# Polyarteritis nodosa presenting as cholecystitis—a case report

**DOI:** 10.1093/jscr/rjad603

**Published:** 2023-11-10

**Authors:** Osama A A Elhardello, Mohammad N Athamnah, Rajaguru Paramaguru

**Affiliations:** General Surgery, Al Salam Al Assima Hospital, Bneid AL Gar, Port Said street, 35151, Kuwait; General Surgery, Al Salam Al Assima Hospital, Bneid AL Gar, Port Said street, 35151, Kuwait; Histopathology, Al Salam Al Assima Hospital, Bneid AL Gar, Port Said street, 35151, Kuwait

**Keywords:** polyarteritis nodosa, gall bladder vasculitis

## Abstract

Medium and small arteries are mainly affected by polyarteritis nodosa. Lungs are spared but any other organ can be involved. Gallbladder can be part of this systemic disease. Isolated gallbladder disease is not common. The presentation of the systemic polyarteritis nodosa as acute cholecystitis is described in this case report. Management of the disease depends on the involved organs and usually consists of systemic steroids. The diagnosis of polyarteritis nodosa should be considered in patients with previous systemic symptoms who develop picture of acute cholecystitis.

## Introduction

Polyarteritis nodosa (PAN) is a systemic necrotizing vasculitis of unknown etiology, mainly affecting medium and small arteries [1]. Virtually, any organ can be affected, but for unexplained reasons the lungs are almost always spared. PAN usually presents with systemic nonspecific symptoms, including fatigue, fever, and weight loss. Skin, joints, GI tract, and kidneys are commonly affected [2].

Systemic vasculitis involving abdominal organs usually runs an aggressive course with a poor prognosis [3]. Gallbladder Vasculitis (GB-V) has been reported as part of a systemic vasculitis or a single-organ vasculitis, which is rare. Differences between the two pathologies are described in clinical presentation, biochemical profile, histological characteristics, course of treatment, and outcomes. In GB-V associated with systemic vasculitis, the clinical presentation is more impressive with significant systemic symptoms, and the treatment is always systemic including the use of immune-suppressants. With single-organ GB-V the presentation is mild, and the treatment is limited to cholecystectomy. Other forms of vasculitis affecting the GB are hepatitis B associated vasculitis, auto-immune vasculitis, and giant cell arteritis.

It’s been reported that in all patients where GB vasculitis occur as part of a systemic vasculitis, treatment with glucocorticoids with or without immunosuppressive agents is needed. It is also reported that the mortality in these patients could reach up to 26% [3].

Although rare, PAN related acute segmental necrotizing pancreatitis and acute appendicitis were reported in the literature [4] [5] . Here, we are reporting a case presented with a clinical and radiological picture of acute cholecystitis that was proven to be PAN on histological examination of the resected GB.

## Case presentation

This is a 61-year-old gentleman with a background of pulmonary fibrosis, essential hypertension, chronic kidney disease, and type 2 diabetes mellitus. He presented with right upper quadrant pain, nausea but no vomiting.

Clinical examination revealed a systemically stable patient with normal temperature. Abdomen was soft and lax but tender at the right upper quadrant with a positive Murphy’s sign.

The biochemical profile of the patient showed a picture of type 1 acute kidney injury and high inflammatory markers. The inflammatory markers have slightly improved, but the renal function continued to deteriorate over time.

Ultrasound scan of the abdomen showed a thickened gallbladder wall of 5.5 mm with some biliary mud.

**Figure 1:**
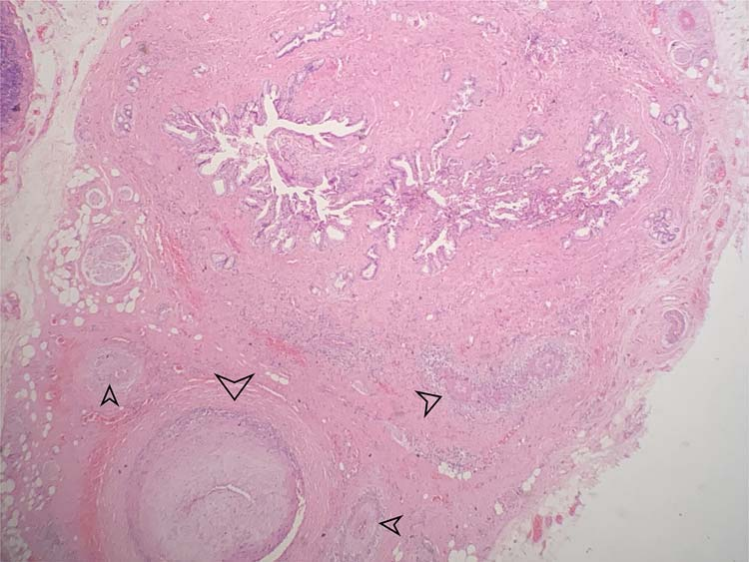
Low power view showing the mucosa of the gallbladder with multiple small and medium sized blood vessels shown by arrows (H&E 40X).

**Figure 2:**
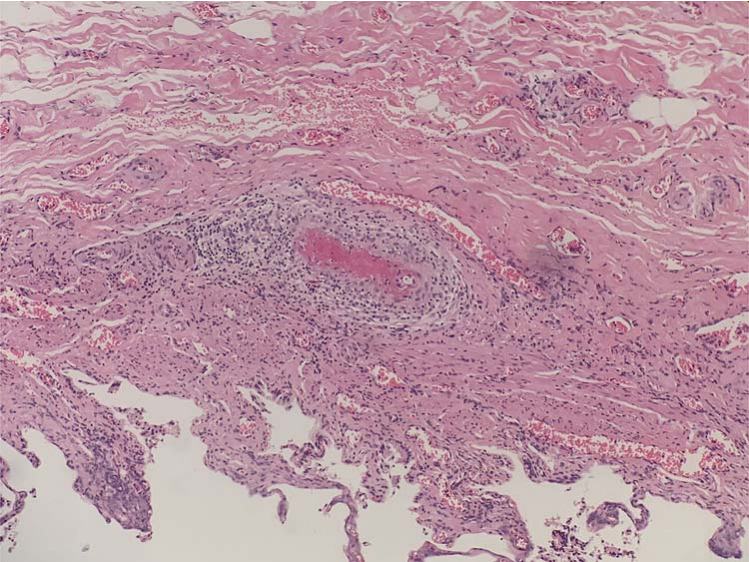
Intraluminal thrombi, fibrinoid necrosis with perivascular infiltration by lymphocytes, neutrophils, and eosinophils (H&E 200X).

**Figure 3:**
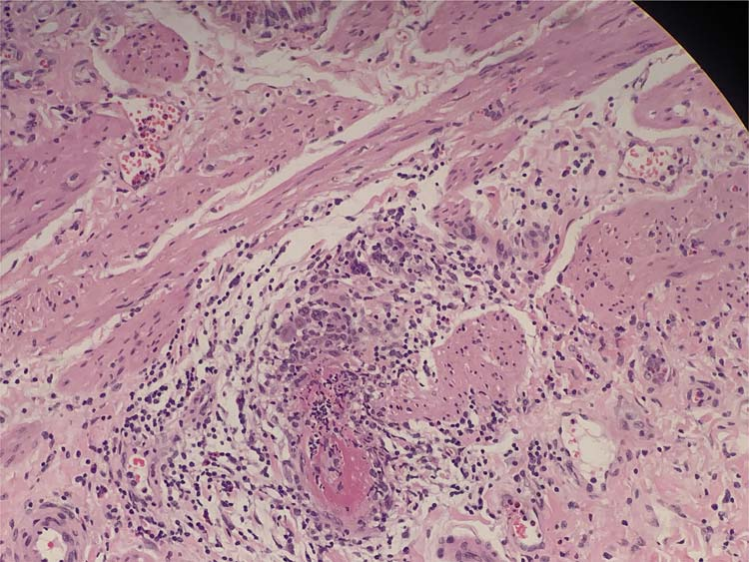
Intraluminal thrombi, fibrinoid necrosis with perivascular infiltration by lymphocytes, neutrophils, and eosinophils (H&E 200X).

He underwent laparoscopic cholecystectomy under general anesthesia in the standard way. The gallbladder was found to be macroscopically inflamed with few omental adhesions. The procedure was completed peacefully, and the patient recovered well. The histology of the gallbladder specimen came back showing features of necrotizing vasculitis of small and medium arteries suggestive of PAN (Figs 1–3). He was treated with steroids for his systemic vasculitis and has shown significant improvement in his symptoms. His kidney function also markedly improved.

On his visit 3 months after the procedure, that patient was symptom free and was only on follow up with the rheumatology and renal teams.

## Discussion

In this report, we present a rare case of PAN that presented clinically with acute cholecystitis in a patient in his sixth decade. Through history and examination, we note that there are symptoms and signs of a systemic disease, such as fever, malaise, and weight loss, with the radiological evidence of acute cholecystitis.

We learnt from the case the possibility of acute cholecystitis to be the initial presentation in patients with systemic vasculitis. This has been reported before, though rare. Our case further confirms the fact that cholecystitis in the presence of systemic vasculitis runs a more aggressive course. In 2014, Rodriguez *et al.* [3] published an analysis of 61 patients comparing between single-organ involvement of the gallbladder and involvement as part of systemic vasculitis. Our case coincided with their findings in patients with systemic vasculitis in terms of similar clinical presentation and histological features. The impressive other feature of systemic vasculitis in our patient was the renal dysfunction, which has only improved after the diagnosis was established and the patient’s treatment with steroids.

Although rare, the presence of systemic symptoms associated with acute cholecystitis picture without gall stone should raise a suspicion to the diagnosis of PAN. As the management could be different. Isolated Single-organ cholecystitis is usually treated with cholecystectomy alone, while in systemic disease and gall-bladder involvement, treatment consists of cholecystectomy together with steroids and immunosuppressants to control the systemic disease.

The patient improved significantly after he was started on high dose of corticosteroids and oral cyclophosphamide.

## Data Availability

Data related to this study is available on request from the corresponding author, Dr. Mohammad Athamnah.
